# Ray propagation imaging and optical quality evaluation of different intraocular lens models

**DOI:** 10.1371/journal.pone.0228342

**Published:** 2020-02-04

**Authors:** Hyeck Soo Son, Grzegorz Labuz, Ramin Khoramnia, Patrick Merz, Timur M. Yildirim, Gerd U. Auffarth

**Affiliations:** The David J. Apple International Laboratory for Ocular Pathology and International Vision Correction Research Centre (IVCRC), Department of Ophthalmology, University of Heidelberg, Heidelberg, Germany; Nicolaus Copernicus University, POLAND

## Abstract

**Purpose:**

Ray propagation visualization and optical performance analysis of four different intraocular lenses (IOLs)

**Methods:**

In this laboratory study, four IOLs with different optical designs were assessed: a monofocal AcrySof IQ SN60WF [Alcon], a diffractive-refractive bifocal AcrySof IQ Restor SN6AD1 [Alcon], a diffractive trifocal AcrySof IQ PanOptix TFNT00 [Alcon], and a diffractive extended-depth-of-focus (EDOF) Symfony ZXR00 [Johnson&Johnson]. An experimental set-up with a water bath containing 0.01% fluorescein solution and monochromatic green laser light (532 nm) was used to visualize the propagation of light rays. Also, the optical performance of the IOLs was evaluated by measuring the modulation transfer function (MTF) values at a pupil sizes of 3.0 and 4.5 mm on the optical bench OptiSpheric^®^ IOL PRO II (Trioptics GmbH, Germany).

**Results:**

Both the diffractive-refractive bifocal IOL and the EDOF IOL showed two defined foci for distance and near vision. In the diffractive trifocal IOL, three distinct foci for distance, intermediate, and near vision could be visualized.

**Conclusions:**

The ray propagation visualization technique allows a qualitative assessment and comparison of light energy distribution between different IOL models. The measured Through-Focus Response (TFR) quantitatively confirmed the evaluated ray propagation behavior.

## Introduction

In recent years, various novel intraocular lens (IOL) models have been developed for the treatment of cataract and presbyopic patients. While monofocal IOLs still constitute the majority of the lenses implanted worldwide [1], multifocal IOLs (MIOLs) are swiftly gaining momentum, largely thanks to their ability to reduce spectacle dependence by generating functional vision not only in far, but also in intermediate and near distances [2].

Such MIOLs use different optical principles to distribute incident light rays to more than one focal point [3], with more recent ones utilizing combined diffractive-refractive, segmental-refractive, or small aperture designs to achieve multifocality. Furthermore, MIOLs that are refined with lathing techniques can minimize the intrinsic drawbacks of MIOLs by enhancing contrast sensitivity and reducing the perception of photic phenomena [4].

Although such IOLs categorically share a multifocal nature, they do differ in their optical quality [5–9] and light distribution behavior [10–12], which are dictated by the optical concept and material composition they employ. Understanding their nature and differences in relation to pupil size may help surgeons in choosing the appropriate IOL design for the individual patient. In this study, we used a dedicated experimental set-up to qualitatively visualize and assess the ray propagation behavior of different multifocal lens models at 3.0 and 4.5 mm pupil sizes.

## Materials and methods

### Intraocular lenses

The following IOLs were analyzed: a monofocal AcrySof, a bifocal AcrySof IQ Restor SN6AD1, a trifocal AcrySof IQ PanOptix TFNT00 (all three lenses from Alcon, Fort Worth, USA), and an extended-depth-of-focus (EDOF) TECNIS® Symfony ZXR00 (Abbott Medical Optics, Santa Ana, USA). Table 1 summarizes the key characteristics of the studied lenses.

**Table 1:** Key characteristics of the tested intraocular lenses.

Optical characteristics of the studied intraocular lenses				
	AcrySof IQ SN60WF	AcrySof IQ Restor SN6AD1	AcrySof IQ PanOptix TFNT00	Symfony ZXR00
Optic Design	one-piece	one-piece/combined diffractive-refractive bifocal	one-piece/combined diffractive-refractive trifocal	one-piece/combined diffractive-refractive extended-depth-of-focus
Total Lens/Optic Diameter	13.0/6.0 mm	13.0/6.0 mm	13.0/6.0 mm	13.0/6.0 mm
Base Power	+21.0 D	+21.0 D	+21.0 D	+21.0 D
Dioptric Power Addition	-	+3.0 D Near Addition	+2.17 D Intermediate Addition +3.25 Near Addition	+1.75 D Near Addition
Lens Material	Hydrophobic Acrylate/Methacrylate Copolymer	Hydrophobic Acrylate/Methacrylate Copolymer	Hydrophobic Acrylate/Methacrylate Copolymer	Hydrophobic Acrylic
Refractive Index	1.55	1.55	1.55	1.47
Spherical Aberration	-0.20 μm	-0.10 μm	-0.10 μm	-0.27 μm

The aspheric AcrySof IQ Restor SN6AD1 has a refractive base dedicated to refracting incident light rays to far focus and a diffractive grating that creates a secondary (near) focus. The diffractive design features apodization that further amplifies distance vision with increasing pupil size by decreasing the diffractive step-height towards the periphery.

The AcrySof IQ PanOptix TFNT00 features a central refractive-diffractive area of 4.5 mm in diameter that is encircled by a refractive ring in the periphery. According to the manufacturer, the diffractive zone incorporates a quadrifocal optic with three unsequential diffraction orders distributing light rays to far, intermediate, and near foci, while the fourth-order further reinforces far vision.

The TECNIS® Symfony ZXR00 is based on a proprietary echelette design with a diffractive-refractive surface that is intended not only to generate the EDOF but also to counteract the chromatic aberration and thereby improve contrast sensitivity.

All studied lenses share an equal base power of +21.0 D.

### Optical quality evaluation

The optical performance of the IOLs was evaluated using the optical bench OptiSpheric IOL Pro II (Trioptics GmbH, Germany), as described in previous studies [13–19]. Its measurement principles adhere to the guidelines governed by the International Standard Organization (ISO) 11979–2 [20] and 11979–9 [21,22] and it thus includes a model cornea (spherical aberration: 0.28 μm) and a model eye containing a balanced salt solution with a refractive index of 1.336 at 25°C.

### Optical quality parameters

The Modulation Transfer Function (MTF) values were measured to analyze the optical performance of the lenses in vitro. MTF is a parameter widely validated and assessed to characterize the optical quality of IOLs objectively [23–26]. In short, the optical bench tests the ability of an optical system to reproduce an infinitesimally thin cross-slit image. The cross-sectional intensity profile of the reproduced image is then calculated into MTF values via the Fourier transform of the Line Spread Function. For both 3.0 and 4.5 mm pupil sizes, the MTF values were measured at 546 nm wavelength with two perpendicular slits corresponding to the sagittal and tangential planes. For the purpose of this study, an average MTF value from the sagittal and tangential plane measurements were used for the analysis.

In addition, a Through-Focus Response (TFR) was also performed along the focal planes of the IOL at a spatial frequency of 50 lp/mm, which corresponds to Snellen visual acuity value of 20/40, to illustrate the IOL’s performance along its focal plane. Depending on the optical design of the studied lens, its TFR may contain one (monofocal) or multiple peaks (multifocal).

### Ray propagation imaging

**Fig 1:**
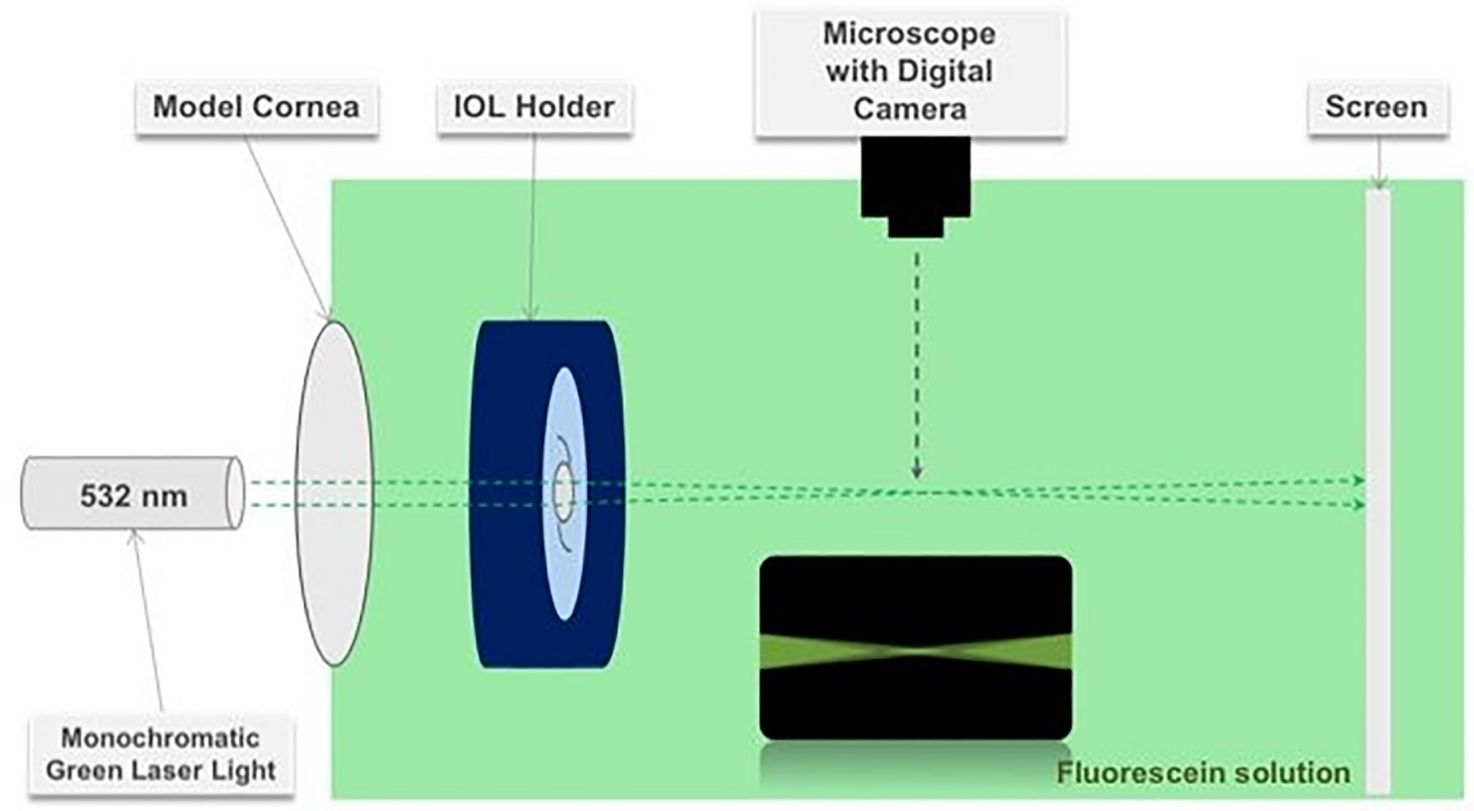
Optical bench set-up for visualization of the ray propagation (not to scale).

In an experimental set-up, each IOL was placed in a lens holder that was submerged in a water bath (1 L) with fluorescein solution (Fig 1): we used a red-orange fluorescein solution (Alcon, Inc., Fort Worth, TX, USA) for injection with 100 mg/mL concentration of fluorescein loaded from a 5 mL glass vial. A monochromatic light beam was then projected through a model cornea (f = 30 mm) and the IOL under test. Although the model cornea used in this study has less power (33.3 D) than that of the human cornea (approx. 43 D) [27], this model is adequate for objects at infinity [20]. The ISO standard describes two model corneas, one is a single lens, and the other is an achromat, with the focal length of 39 mm and 32 mm, respectively, which is close to the cornea lens used in the current study. For the purpose of this study, a green laser light (532 nm) was used. Although a 546-nm laser would match the light conditions of the optical quality measurements precisely, this was not available. However, the wavelength difference of 14-nm between the light sources of the two experimental setups seems negligible, given the visualization purpose of the ray-propagation analysis. The selection of the green light may also be advantageous for two reasons. 1) The optical design and energy efficiency of most diffractive IOLs are optimized for the green light. 2) The human eye is most sensitive to yellowish-green color under standard light conditions with its sensitivity peak at approximately 555 nm [28]. The visualized ray propagation was then captured with a digital camera mounted on a surgical microscope (Leica, Wetzlar, Germany) using a 40x magnification. The ImageJ program, a Java-based image-processing software provided by the US National Institute of Health, was used to obtain pixel intensity values along the optical axes. Following conversion from a pixel unit to mm using a scale bar, the later was converted to a defocus range (in diopters) based on the thin lens approximation (S1 File).

## Results

**Fig 2:**
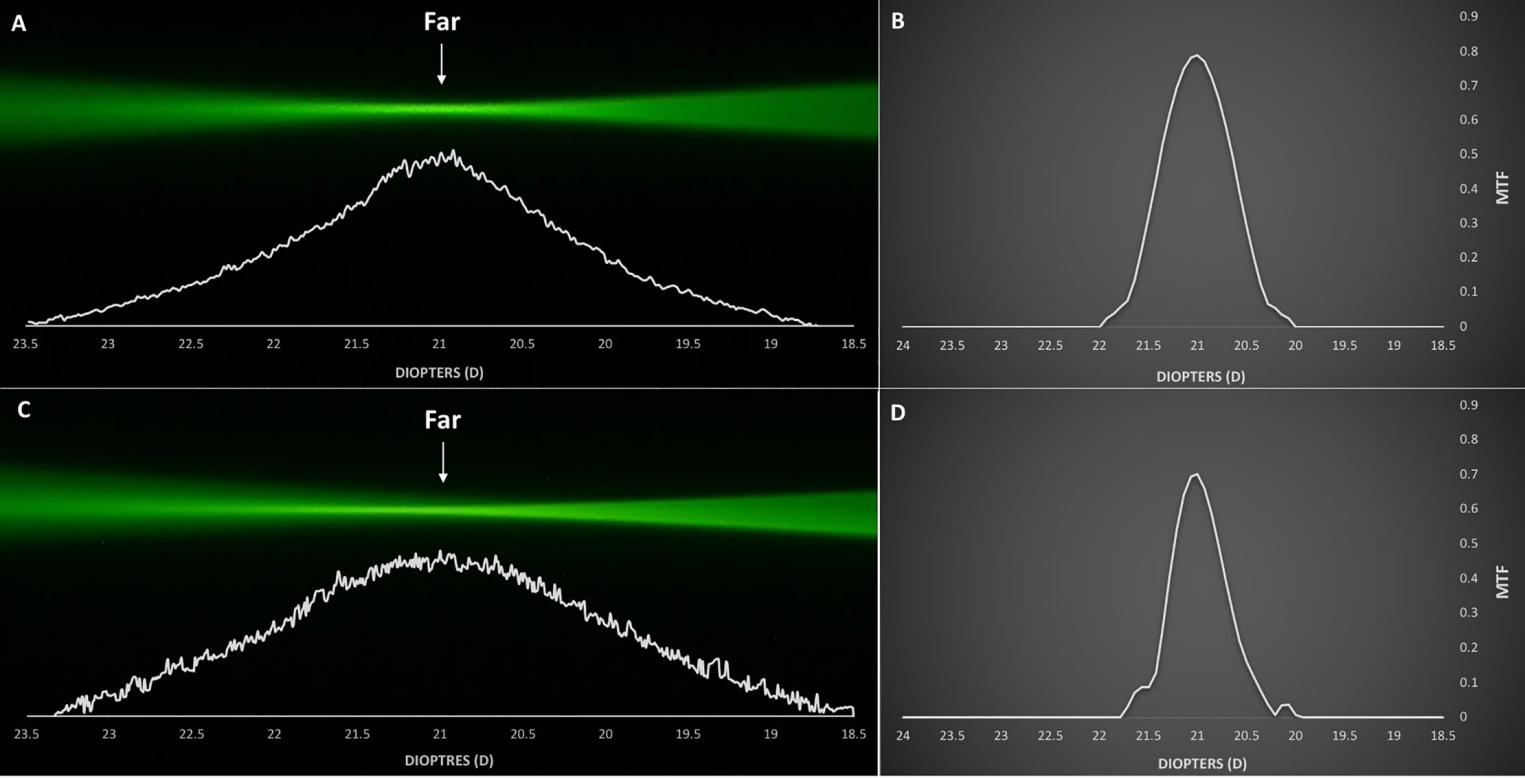
Optical ray propagation and Through-Focus Response of the AcrySof IQ Monofocal IOL at 3.0 mm (A, B) and 4.5 mm (C, D) pupil sizes.

**Fig 3:**
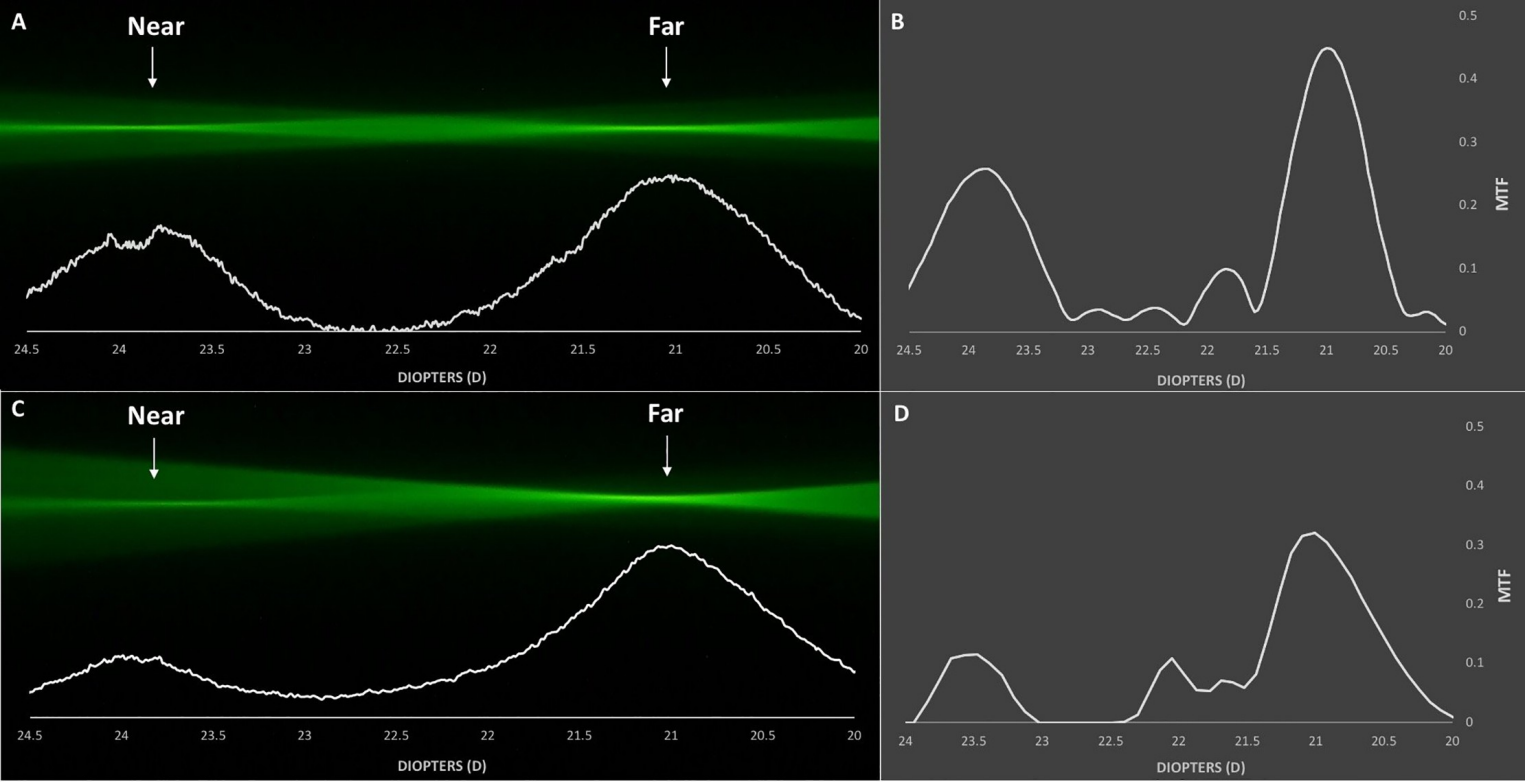
Optical ray propagation and Through-Focus Response of the AcrySof IQ Restor SN6AD1 at 3.0 mm (A, B) and 4.5 mm (C, D) pupil sizes.

**Fig 4:**
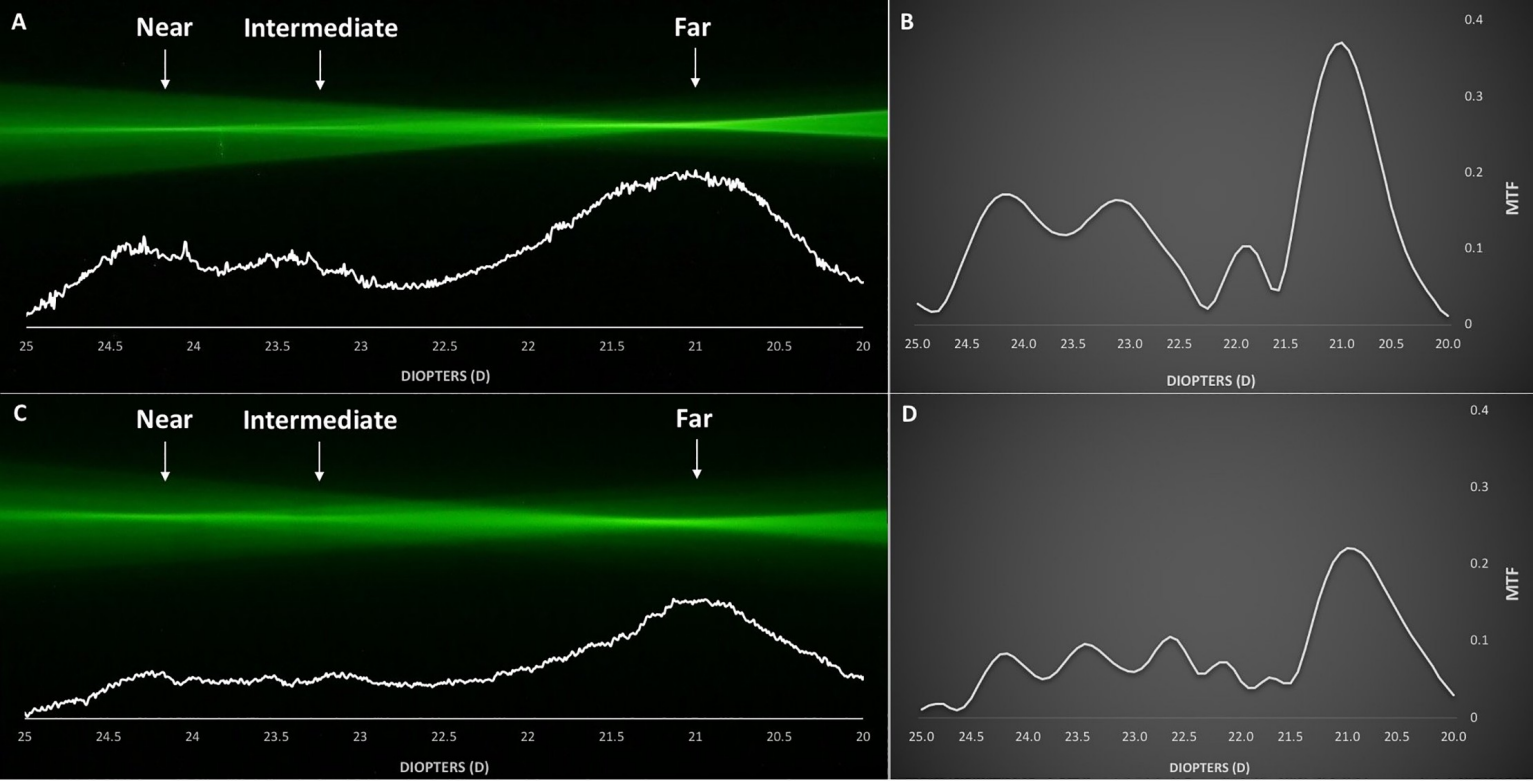
Optical ray propagation and Through-Focus Response of the AcrySof IQ PanOptix TFNT00 at 3.0 mm (A, B) and 4.5 mm (C, D) pupil sizes.

**Fig 5:**
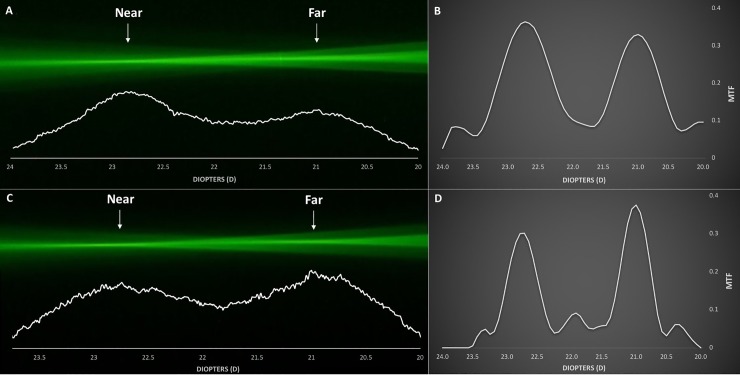
Optical ray propagation and Through-Focus Response of the TECNIS Symfony ZXR00 at 3.0 mm (A, B) and 4.5 mm (C, D) pupil sizes.

Figs 2–5 demonstrate the ray propagation and TFR of the tested IOLs at apertures of 3.0 and 4.5 mm. The images were taken at the same magnification power. The white graphs directly below the visualized optical ray propagation reflect the distribution of light energy as measured by the intensity of the pixel values along the optical axes. Table 2 shows the MTF values measured at a spatial frequency of 50 lp/mm for 3.0 and 4.5 mm pupil sizes.

**Table 2:** Measured MTF values at a spatial frequency of 50 lp/mm for 3.0 and 4.5 mm pupil sizes.

	IQ		Restor		PanOptix		Symfony	
Pupil Size (in mm)	3.0	4.5	3.0	4.5	3.0	4.5	3.0	4.5
Far	0.790	0.701	0.450	0.321	0.371	0.221	0.330	0.376
Intermediate	-	-	-	-	0.164	0.106	-	-
Near	-	-	0.259	0.114	0.172	0.084	0.364	0.302

AcrySof IQ Monofocal IOL (Fig 2):

At both pupil sizes, the incident light rays are refracted to a single focal point. The TFR only shows a slight decrease in the MTF value at 4.5 mm pupil size (MTF = 0.701) compared to the value at 3.0 mm (MTF = 0.790).

AcrySof IQ Restor SN6AD1 (Fig 3):

Both the pixel values obtained from the ray propagation as well as the TFR show two clear peaks at 3.0 mm pupil size, with a higher amount of light energy allocated to the far (MTF = 0.450) than to the near (MTF = 0.259) focus. With increasing pupil size, the Restor IOL exhibited an even more distance-dominant light distribution behavior, with MTF value for far focus (MTF = 0.321) reaching almost three-fold of that for near focus (MTF = 0.114). This effect of apodization could also be confirmed via a ray-propagation evaluation.

AcrySof IQ PanOptix TFNT00 (Fig 4):

The ray propagation of the PanOptix TFNT00 demonstrated three distinct foci, each for far, intermediate, and near focus, at both 3.0 and 4.5 mm pupil sizes. As displayed by the light energy distribution pattern of the ray propagation and TFR for 3.0 mm aperture, PanOptix TFNT00 allocated the highest amount of light energy to the far focus (MTF = 0.371), followed by the near (MTF = 0.172) and intermediate focus (MTF = 0.164). Also, for a 4.5 mm aperture, the far focus (MTF = 0.221) obtained the most light energy compared to the near (MTF = 0.084) or intermediate (MTF = 0.106) focus.

TECNIS® Symfony ZXR00 (Fig 5):

At a 3.0 mm aperture, Symfony ZXR00 allocated more light energy to the near (MTF = 0.364) than to the far (MTF = 0.330) focus, while it became more far-dominant at a 4.5 mm aperture (MTF = 0.376 for far focus and MTF = 0.302 for near focus).

## Discussion

The ray propagation behavior of different IOL models could successfully be visualized qualitatively and assessed quantitatively using the proposed imaging technique. As it does not require specialized optical components, this technique can be readily adopted to assess the light energy distribution of an IOL in relation to pupil size and quantify basic optical parameters, e.g., the nominal and add power, for both research and educational purposes.

The light energy distribution graph derived from the pixel intensity profile confirmed the fundamental optical properties of the studied IOLs. The monofocal Acrysof IQ showed incident light rays being concentrated to a single focal point (Fig 2). The Acrysof IQ Restor SN6AD1 demonstrated two separate foci that correspond to far and near vision and a characteristic change in light energy allocation. The energy distribution of the Restor is innate to its optical design that employs apodization, which was successfully visualized as the far-to-near ratio of light energy distribution shifted in favor of far focus with increasing pupil size (Fig 3). The AcrySof IQ PanOptix TFNT00 demonstrated a trifocal light energy propagation, allocating most of its incoming light rays to the far focus, followed by the near and intermediate foci (Fig 4). The diffractive Symfony ZXR00, though reported as an EDOF lens, showed a more bifocal ray propagation behavior. The light energy intensity profile reflects how the two foci are separated by the near addition dioptric power of the lens. While it acted slightly more near-dominantly at the 3.0 mm aperture, the larger pupil size reversed the light energy allocation, distributing more light rays to the far focus (Fig 5).

Our light intensity profile results were visually comparable to the TFR calculated by the fully automated optical test system. This apparent similarity indicates the importance of the energy distribution to the image quality of IOLs. However, as two different model corneas were used for the two measurement set-ups, a direct comparison is possible only on a limited scale. Furthermore, other factors (e.g., aberrations, refractive index) can also influence the optical quality, so one cannot conclusively infer the quality of the IOL solely based on its ray-propagation behavior. Moreover, the ray-propagation set-up is significantly affected by light scattering, which on the one hand enables to visualize the rays, but on the other hand, causes the loss of light at the primary and secondary focus affecting the light intensity profile.

Spherical aberration is a monochromatic phenomenon that occurs when light rays do not converge to the ideal (Gaussian) focus, but their intersection with the optical axis changes with the ray height. In a young eye, the positive spherical aberration of the cornea is usually offset by the negative spherical aberration of a clear crystalline lens. With increasing age, the negative lenticular spherical aberration becomes gradually positive in relation to cornea’s rather stagnant positive value, thereby disrupting the subtle balance of spherical aberration between the cornea and the lens might lead to deteriorated visual quality [29,30]. In 2008, Terwee et al. used an experimental set-up to evaluate the ray propagation behavior of different spherical and aspheric intraocular lens models and qualitatively assessed the impact of spherical aberration on their optical quality [8]. Using United States Air Force (USAF) 1951 resolution test target images and the MTF assessment, they showed that aspheric IOLs that fully compensate for the positive corneal spherical aberration demonstrate the highest optical performance regardless of pupil size, while spherical lenses with no spherical aberration correction show the lowest optical quality that degrades with increasing aperture [8]. They reported that at the far focus under mesopic conditions, the retinal image of an aspheric multifocal IOL even outperforms that of a spherical monofocal IOL. All IOLs assessed in our study have aspheric designs. However, they differed in their level of SA correction, which ranged from -0.10 μm in the Restor and PanOptix IOLs to -0.27 μm in the Symfony (Table 1). Given that asphericity of the Symfony IOL almost entirely compensated for 0.28 μm SA of the model cornea, this lens did not suffer a significant deterioration of the optical quality when the aperture size increased. A good performance of the Symfony IOL at the 4.5 mm pupil contrasts with a 40% MTF loss (@50 lp/mm) at the far focus of the PanOptix, which only partially corrects SA of the model cornea, indicating the importance of SA correction in the assessment of IOL optical quality.

Although the optical quality of IOLs has typically been assessed in monochromatic green light, the *in vivo* performance of the IOL is also affected by material dispersion while function in the polychromatic light. Therefore, when comparing the optical quality of different IOLs, it is also important to consider the Abbe number, which quantifies the material dispersion. Optical materials characterized by a lower Abbe number have greater chromatic dispersion, which results in longitudinal chromatic aberration (LCA) [31]. LCA, in short, describes the inability of an optical system to refract incident rays of different colors onto the same focal plane. In a standard refractive system, the focal points of colors with shorter wavelengths lie before those of colors with longer wavelengths [32]. However, the reverse is the case when LCA is produced by a diffractive lens. Of the IOLs analyzed in this study, AcrySof IQ SN60WF, AcrySof IQ Restor SN6AD1, and AcrySof IQ PanOptix TFNT00 share an Abbe number of 37, while Symfony ZXR00 has an Abbe number of 55. In addition to the lower dispersion of the Symfony, this lens features a chromatic-aberration correction, which may further enhance its polychromatic performance. On the other hand, diffractive technology, as is implemented in the Symfony lens, has shown strong spectral dependency [33,34]. A similar effect was found in other IOLs utilizing diffractive gratings because the diffraction efficiency changes if wavelength different from the one it is designed for is used (e.g., 555 nm) [33–36]. A recent clinical study has shown that this dependence of the optical performance on wavelength may affect the patient's quality of vision [35]. Future studies assessing the differences in the optical quality between these IOLs under polychromatic light may help to elucidate their performance and more closely mimic real-life situations.

To summarize, our imaging technique presents an easily accessible modality to visualize the light energy distribution of different IOL models directly. This method may be helpful to researchers, surgeons, and their patients in understanding the optical properties of multifocal IOLs and examining the trajectory course of incident light rays with varying pupil sizes. Further *in vitro* studies evaluating the ray propagation of IOLs under polychromatic light or light distribution of opacified IOLs, which are known to cause light scatter, may also provide valuable information about their nature *in vivo*.

## Supporting information

S1 File Raw data for pixel intensity and modulation transfer function values in relation to power for each intraocular lens and pupil size. (XLSX)

S1 Appendix Thin lens approximation. (DOCX)
