# Correction: Ray propagation imaging and optical quality evaluation of different intraocular lens models

**DOI:** 10.1371/journal.pone.0233829

**Published:** 2020-05-21

**Authors:** Hyeck Soo Son, Grzegorz Labuz, Ramin Khoramnia, Patrick Merz, Timur M. Yildirim, Gerd U. Auffarth

The Funding statement is incorrect. The correct Funding statement is as follows: This study was supported by the Klaus Tschira Foundation, Heidelberg, Germany.
