# Supplementary material for: Ray propagation imaging and optical quality evaluation of different intraocular lens models
Source: PLoS One. 2020 Feb 4;15(2):e0228342. doi: 10.1371/journal.pone.0228342 (PMC6999873; doi:10.1371/journal.pone.0228342)
Supplement: S1 Appendix — (DOCX) [file pone.0228342.s002.docx]

**S1 Appendix**

The focal length (*f*) of an optical system comprised of two elements can be derived from the thin-lens formula:

$\frac{1}{f}=\frac{1}{f_{c}}+\frac{1}{f_{IOL}}-\frac{d}{{n\cdot f}_{c}{\cdot f}_{IOL}}$ Eq. 1

Where *f_c_* and *f_IOL_* are the focal lengths of the cornea lens and the IOL, respectively. The *d* parameter is the distance between the two lenses.

The optical power (*Φ*) of this two-lens system in a medium with the refractive index *n* is the inverse of its focal length:

$\text{φ}=\frac{1}{f}$ Eq. 2

The diopter-power change with respect to the IOL’s far focus can be calculated as the difference between the optical power (Φ) and the estimated power after the shift by 1 mm along the optical axis (Φ_S_). The power change per mm (Φ_per mm_) is then expressed as:

${\text{φ}_{per mm}\text{= φ}}_{S}-\text{φ}$ Eq. 3
